# Conservative method for vertical electrooculogram attenuation based on local suppression of ongoing EEG artifact templates

**DOI:** 10.1371/journal.pone.0305902

**Published:** 2024-07-18

**Authors:** Dimitri Marques Abramov, Paulo Ricardo Galhanone, Vladimir V. Lazarev, Antonio Mauricio Ferreira Leite Miranda de Sá

**Affiliations:** 1 Laboratory of Neurobiology and Clinical Neurophysiology, National Institute of Women, Children, and Adolescents Health Fernandes Figueira (IFF), Fundação Oswaldo Cruz (FIOCRUZ), Rio de Janeiro, RJ, Brazil; 2 Biomedical Engineering Program, Federal University of Rio de Janeiro, Rio de Janeiro, RJ, Brazil; Al-Nahrain University, IRAQ

## Abstract

Eye movement during blinking can be a significant artifact in Event-Related Potentials (ERP) analysis. Blinks produce a positive potential in the vertical electrooculogram (VEOG), spreading towards the posterior direction. Two methods are frequently used to suppress VEOGs: linear regression to subtract the VEOG signal from the electroencephalogram (EEG) and Independent Component Analysis (ICA). However, some information is lost in both. The present algorithm (1) statistically identifies the position of VEOGs in the frontopolar channels; (2) performs EEG averaging for each channel, which results in ’blink templates’; (3) subtracts each template from the respective EEG at each VEOG position, only when the linear correlation index between the template and the segment is greater than a chosen threshold L. The signals from twenty subjects were acquired using a behavioral test and were treated using FilterBlink for subsequent ERP analysis. A model was designed to test the method for each subject using twenty copies of the EEG signal from the subject’s mid-central channel (with nearly no VEOG) representing the EEG channels and their respective blink templates. At the same 200 equidistant time points (marks), a signal (2.5 sinusoidal cycles at 1050 ms emulating an ERP) was mixed with each model channel and the respective blink template of that channel, between 500 to 1200 ms after each mark. According to the model, VEOGs interfered with both ERPs and the ongoing EEG, mainly on the anterior medial leads, and no significant effect was observed on the mid-central channel (Cz). FilterBlink recovered approximately 90% (Fp1) to 98% (Fz) of the original ERP and EEG signals for L = 0.1. The method reduced the VEOG effect on the EEG after ERP and blink-artifact averaging in analyzing real signals. The method is straightforward and effective for VEOG attenuation without significant distortion in the EEG signal and embedded ERPs.

## 1. Introduction

Vertical eye movement during blinking produces a positive and symmetrical potential on the electroencephalogram signal, the vertical electrooculogram (VEOG), with the most prominent event at frontopolar (Fp) sites (where it is easily observable), which decays by conduction along the scalp surface [1]. In Event-Related Potentials (ERP) studies, such a considerable potential can be a significant artifact that may interfere with waveforms during signal averaging, mainly at anterior scalp sites. If the blink is a reflexive and systematic response to testing design (such as attentional tests that promote expectancy), and it is time-locked to the studied events, even the low amplitude VEOG at posterior channels could reveal a significant effect upon the event-related EEG potentials [2, 3].

Parallel to the development of human neurophysiology, methods for minimizing the VEOG effect on neural signals have been implemented. The digital signal recording has allowed several offline signal processing strategies to suppress VEOG effects. A usual method uses the electrooculogram signal (EOG-S) recorded from the ocular channel (around the eyes) as a template to extract the VEOG after a linear regression between the latter and each channel, and its corresponding correlation coefficient reduces EOG-S amplitude, which is subtracted from the EEG signal [4–6]. However, this method corrupts the neural information as the EOG-S also contains an EEG signal, which is also subtracted by the algorithm [7].

Another method offers more effective and reliable results, although it is computationally more intensive: correction using Independent Component Analysis (ICA) [7, 8]. ICA has become a standard tool for neuroscience data analysis [9]. It was first introduced in the 1980s as a tool for analyzing composite messages produced by a set of sensors, each sensitive to a composition of multiple source signals [10]. This seminal work used an analogy with messages carried out throughout nervous fibers to develop the computational architecture used there. According to the ICA model, a set of observed signals results from a linear mixture of mutually independent sources. Since then, many other ICA algorithms have been proposed, such as the FastICA (Hyvarinen’s fixed point algorithm) [11], Infomax (Information-Maximization) [12], SOBI (Second Order Blind Identification) [13] and JADE (Joint Approximation Diagonalization of Eigenmatrices) [14].

Although such distinct algorithms exploit different features in the signals, e.g., correlation (second-order statistics) structure or higher-order statistics in the data, if one of them is chosen for ICA-based EEG correction, an external classifier will be needed to choose which components refer to the EOG-S [7, 8, 15, 16]. Despite the indicated benefits of ICA in the case of few EEG channels [17], it would have a severe issue concerning eye-blinking artifact removal since it is not fully efficient when there is bidirectional contamination between the desired signal and the artifact. This issue occurs because rejecting the artifact component altogether may introduce a significant distortion in the reconstructed EEG signal [18].

In order to mitigate the problems that might arise due to bidirectional contamination between the signal and artifact, some pre-processing stages have been incorporated, with the discrete Wavelet Transform (WT) [19] being among the most used ones, resulting in the so-called ICA-WT [20]. Wavelet decomposition results in the approximation and detail coefficients. While these first typically capture the broader and more general features of a given signal, the latter capture mainly minor details and noise. Thus, only the approximation or detail with more remarkable similarity to the artifact may have its statistical independence analyzed by ICA, reducing the bidirectional contamination between signal and artifact.

On the other hand, adaptive filtering (AF) [21], which involves applying digital filters whose coefficients change with the aim of making the filter converge to an optimal state, has also been used to remove artifacts in the EEG [22]. Unlike ICA-WT, adaptive filtering does not require pre-processing; instead, it requires a reference signal to be used in the adapting algorithm [23], which may also represent a limitation of the method. This latter aspect may explain why adaptive filtering is less applied in EEG artifact removal than ICA-based methods.

The ICA basic and more sophisticated methods with pre-processing, such as the ICA-WT, and, to a lesser extent, adaptive filtering, may be considered the current state-of-the-art in EEG artifact removal. However, the ICA model assumes that the number of channels should equal or exceed the number of mixing sources [24]. In cases when such a balance between measured signals and sources is not observed, the mixing system is not invertible, and hence, the independent component cannot be obtained straightforwardly. Such cases, often called ICA with overcomplete bases, represent a challenging problem [11].

Therefore, with fewer channels on the scalp, there might be more neural information from different sources in the components than in the artifact signal. Thus, ICA could corrupt the ERPs of interest if the EEG setup has twenty channels (conventional 10–20 system), for example.

Trying to overcome the problem that appears with ICA with overcomplete bases, the present work aims at developing an automatic algorithm for the suppression of VEOG, which is suitable for both the ERP analysis (which works on the time domain with minimum EEG corruption) and for datasets with few channels. We have thus implemented a more straightforward approach that attempts to suppress only the VEOG artifacts from the EEG. The method statistically detects the VEOG positions in the signal from the frontopolar channels (Fp1 and Fp2), where this artifact has the most significant amplitude (up to three times the EEG amplitude). The EEG epochs fixed by the points detected at Fp1/2 are averaged for each channel, yielding a "blink template." This template was subtracted from the respective EEG segments if the correlation coefficient between the template and the segment was greater than a predefined threshold. A non-linear residual signal primarily remains from each VEOG, disappearing in the subsequent averaging.

To evaluate the present method and estimate its best working parameters, a model made from a biological "blink-free" EEG signal (from the mid-central channel) was adopted, where a triggered sinusoidal signal (emulating an ERP) was periodically inserted, followed by a VEOG which partially overlapped that signal. Additionally, data from a forced-choice experimental paradigm to analyze P3 waves (where subjects had usually blinked after their response) were treated using FilterBlink.

## 2. Methods

### 2.1. Experimental procedures and ERP acquisition

Twenty typically developing boys (9–13 y.o.) were recruited for this work after they agreed and their parents gave their written consent. This research was approved by our Institutional Ethics Board under CAAE 08340212.5.0000.5269. These boys submitted to the Attention Network Test [25], a forced choice protocol with two alternatives for evaluation of target orientation (a yellow fish facing right or left), chosen by pressing the respective keyboard arrows. Different kinds of cues indicate the target position in the visual field. Reaction time, answer accuracy, and long latency ERPs, recorded at the parietal and frontal sites, were studied. The ANT is divided into trials with 1650ms of stimulus between cue and target onsets, separated by gaps randomly varying between 1000 and 2000 ms, after the behavioral response. In each trial, the subject was asked to fix their glance at a central cross and answer about the target orientation (left or right) (a yellow fish flanked by two other identical fishes on each side). Each subject performed nine blocks (a training block plus eight test blocks), with 24 trials each (for all possible cues and target conditions).

The setup Neurofax ® 1200 (Nihon Kohden) with 20 channels (10–20 montage system) was used, with Ag-AgCl electrodes over the scalp, referenced at linked earlobe electrodes (A1-A2). The impedances were maintained below 10 kΩ. Target stimuli were synchronized to the EEG by a digital trigger from the computer that displayed the ANT screens and recorded the answers. The neural signal was digitalized with a sampling rate of 1000Hz and 24-bit resolution. High- and low-pass filters were 0.5 and 150 Hz, respectively, with a notch filter at 60 Hz. These filters were applied after acquisition (offline). The remaining relevant noise (e.g., muscular artifacts) was eliminated by manual inspection of the EEG signal before averaging and after FilterBlink application.

### 2.2. The FilterBlink algorithm

The present method aims to suppress the VEOG artifact by subtracting its "template" from an EEG segment when it shows sufficient correlation with the template. It is obtained for each channel by averaging the VEOGs triggered at the frontopolar channels (Fp1 and Fp2, by the 10–20 montage system). The time positions where the blinks occur are detected when the signal amplitude exceeds a threshold given by the mean ($\bar{x}$) of the EEG signal modulus in the Fp1 and Fp2 channels plus the standard deviation (σ) multiplied by a constant k. Therefore, from these EEG signals of size M, the vector of the VEOG positions, *P*, of size *N*, was extracted for EEG amplitude-values greater than $\bar{x}$ + *k* σ (In the present case, k was set to 1.5 based on the heuristic criterion). All points, *p*_*i*_, between *i* and *i + 100ms* have been discarded as they may belong to the same VEOG., resulting in two vectors of wave points (P’) for Fp1 and Fp2. The rate of their lengths is a measure of confidence. If it lay in an arbitrary interval, d (here, 0.9 < d < 1.1), the detection of VEOGs would be regarded as valid. Otherwise, FilterBlink would be aborted.

In the more conservative scenario, the shorter P’ vector is assumed to be the indexing vector of the central positions of the VEOGs, and it is applied to all channels. Hence, the EEG signal for each one of the 20 channels, X, is averaged inside an epoch p’_i_−t and p’_i_ + t, yielding the VEOG templates Θ (vector with amplitude values at the time, for each channel, j, from the channel set, X). If one decides to be less conservative, the more extended P’ vector may be used instead, but this may lead to erroneous VEOG being removed, resulting in a newly added artifact with an inverted signal (i.e., with phase shifted by 180 degrees).

The linear correlation coefficients, *r*, are obtained between the respective VEOG template and the EEG epoch from an EEG channel *X*_*j*_ using Pearson’s method. Whenever *r > L*, the template Θ from the EEG channel *X*_*j*_ is subtracted from the respective epoch.

**Fig 1:**
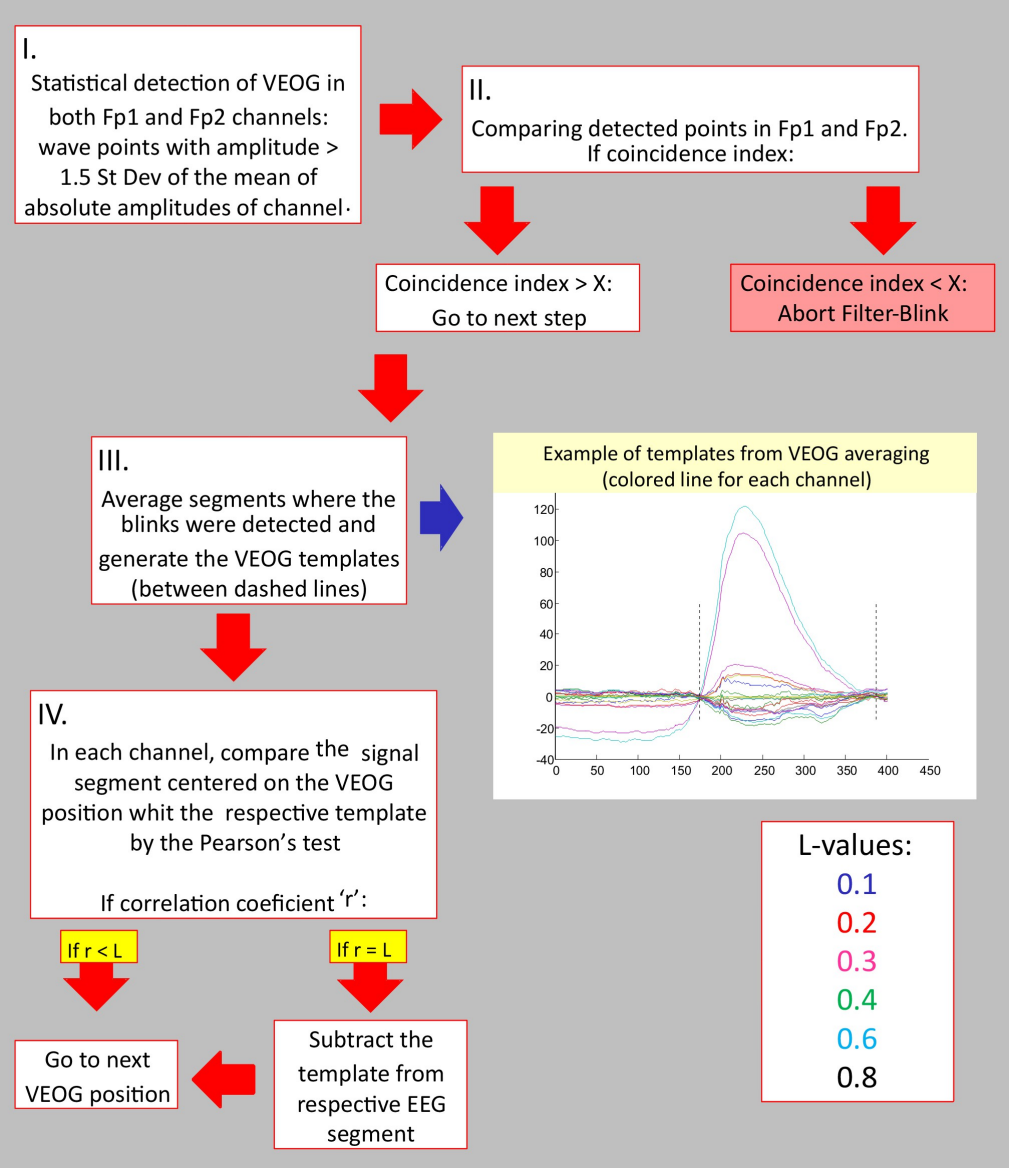
Diagram of the FilterBlink algorithm. See text for details. On the step II, X represents a custom threshold (usually X = 0.9).

The adopted values for *L* are 0.1, 0.2, 0.3, 0.4, 0.6, and 0.8. (See Fig 1 for a diagram explaining the FilterBlink operating algorithm).

### 2.3. The EEG model

**Fig 2:**
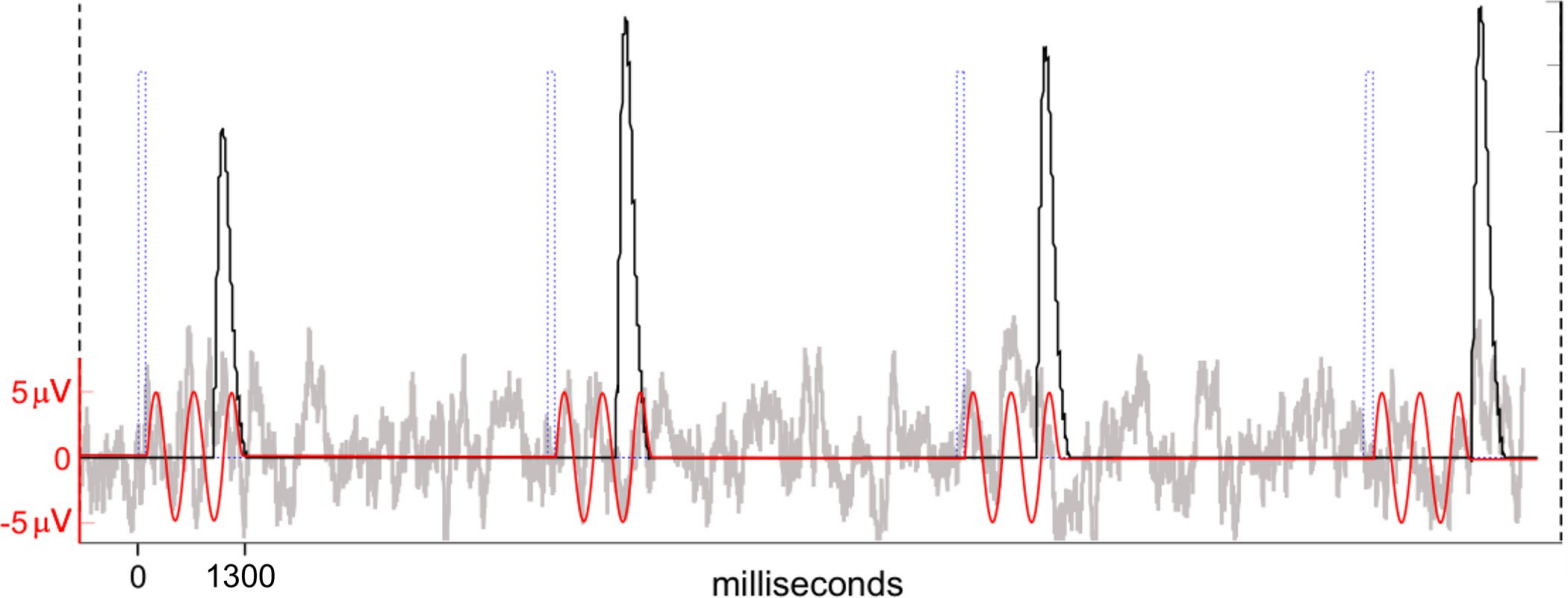
Schematic illustration of the generation of the modeled EEG signal. On the subject’s Cz EEG signal (with smallest natural blink artifact), a known senoid wave 1300ms long (artificial ERP) was added immediately after each trigger pulse of a sequence arbitrarily defined. VEOG templates were also added at semirandom time points, sometimes overlaying the artificial ERPs.

In order to verify its efficacy, the present method was tested using an EEG model with embedded artificial event-related potentials (ERPs) with predetermined parameters. A model was performed for each subject using 20 copies of his Cz signal, where the blink artifact is relatively irrelevant. These 20 copies are the base for the modeled channels, equivalent to those from the 10–20 system. A trigger vector with 200 markers spaced 4400 ms apart was generated. A sinusoidal wave with 2.5 cycles, -5 to 5 μV of amplitude, and 1150ms long was inserted 100ms after every marker onset in all model channels, representing the ERP. The real VEOG templates from the respective subject, per each channel, were added to their respective modeled channels after each marker, with their position randomly ranging from 500 to 1200 ms (the frequency of random values was normally distributed). The amplitude of the VEOG templates was stochastically enlarged up to 1.4 times. See Fig 2.

### 2.4. Statistical analysis

Pearson’s method was used to test correlations between modeled EEG signal without VEOG inclusion (only the artificial ERP wave was embedded), EEG signal with included VEOG artifact, and modeled EEG signal after FilterBlink application for each L value. The correlation coefficients formed a dataset for statistical comparisons between these three conditions using the Wilcoxon Signed Rank Test for paired variables.

The results of FilterBlink application in the real EEG were qualitatively described for the ongoing EEG at frontopolar channels, as well as the effects of FilterBlink on ERP waves and averaged blink artifacts.

## 3. Results

**Fig 3:**
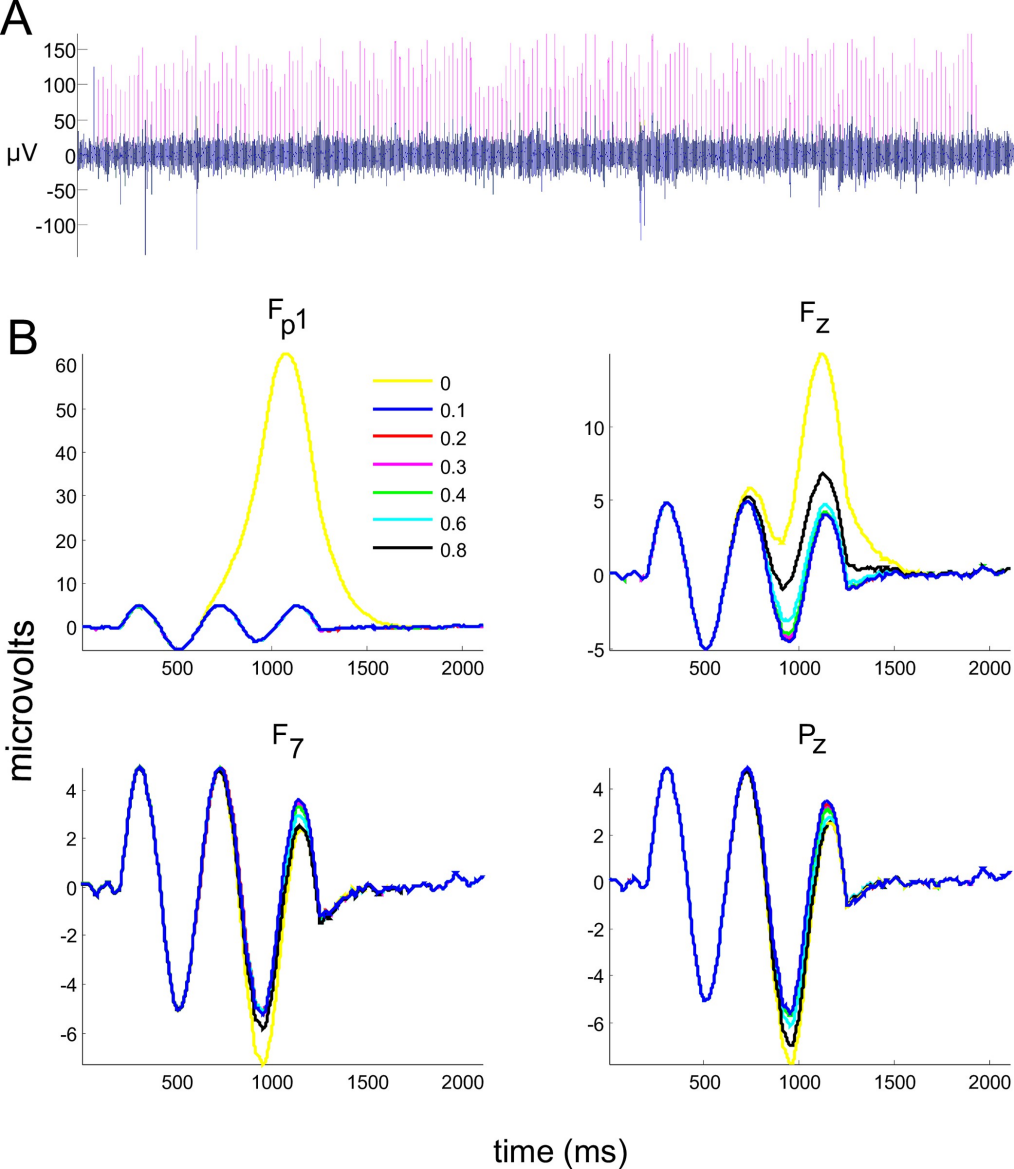
Application of FilterBlink on the EEG modeled signal. (a) Illustrative effect on the Fp1 modeled channel, prior to (magenta) and after (blue) FilterBlink application. (b) After signal averaging is locked to trigger vector, the effect of each L value on FilterBlink application at Fp1, Fz, F7, and Pz modeled channels, showing best ERP (sinusoidal) recovering for L = 0.1 (see color legend). Yellow = without FilterBlink.

The present method apparently eliminated most blink artifacts in the modeled EEG signal from the Fp1 channel (see Fig 3A). The ERPs prior to (Fig 3B, yellow line, for channels Fp1, Fz, F7, and Pz) and after FilterBlink application (colored lines for each L value, Fig 3B) were substantially different despite the fact that signal distortion by VEOGs seems to be more relevant in Fz and frontopolar channels.

**Fig 4:**
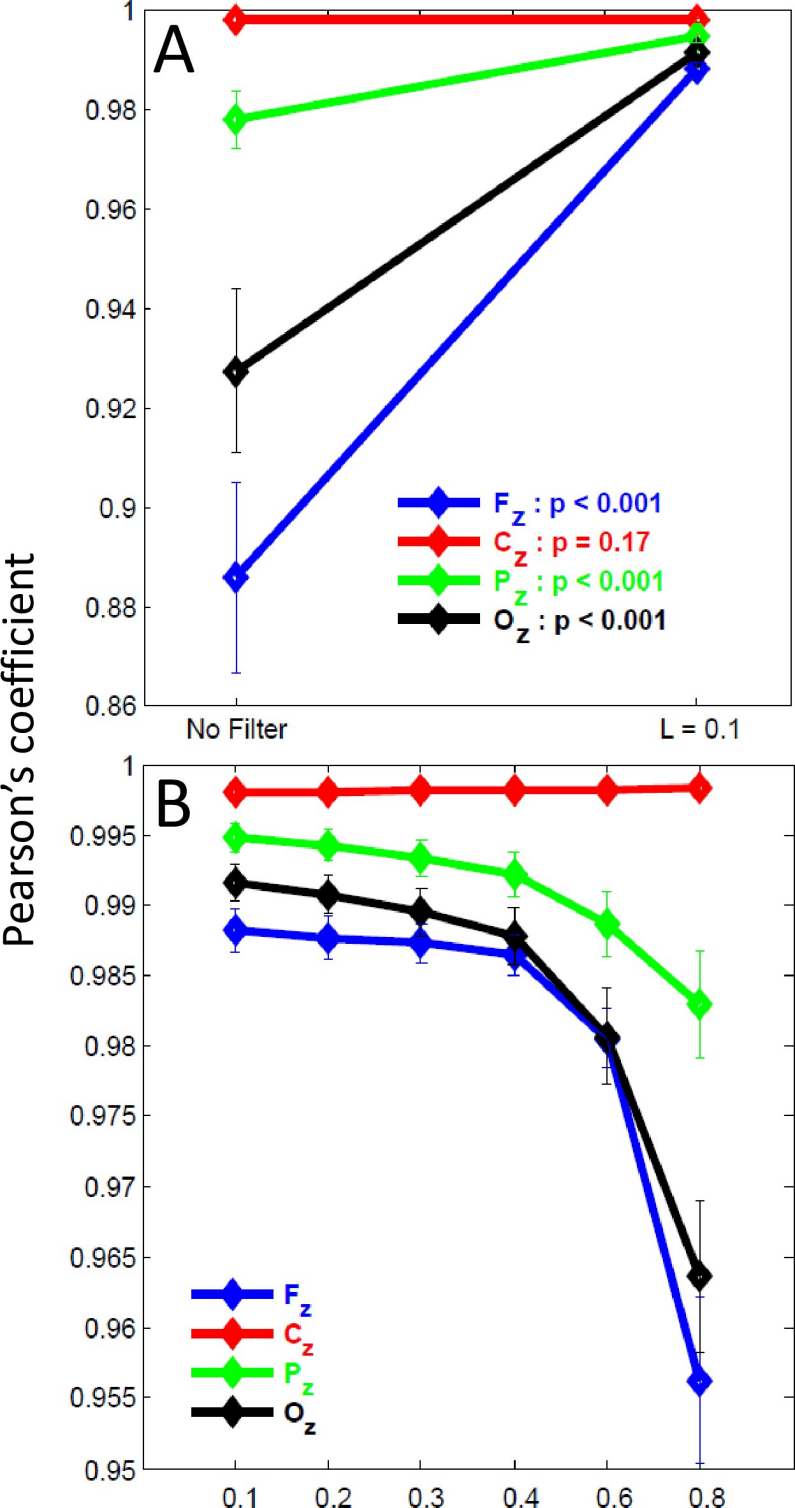
Analysis of the FilterBlink effect on the correlation index between modeled ongoing EEG without VEOGs and modeled ongoing EEG with VEOGs. (a) Correlation index prior to and after FilterBlink application (L = 0.1). (b) Correlation index for different L values (sample mean ± std. dev.).

Paired comparisons of mean correlation indexes of modeled signals with and without VEOG inclusion, prior to and after FilterBlink application (Fig 4A, red line), revealed that the modeled signal at Cz channel had no prominent alteration (p > 0.05, n = 20) and FilterBlink did not alter this correlation for all *L* values (Fig 4B, red line). However, in the other channels, including the VEOG templates, such comparison yielded low correlation coefficients between the signals before and after adding VEOG artifacts (Fig 4A). The FilterBlink application recovered the similarity with the signals before artifact inclusion when coefficients turn from 0.985 to 0.995, for L = 0.1 (p < 0.001, Wilcoxon Rank Test, for Fz, Pz and Oz, Fig 4A). The higher L values yielded a progressive reduction of similarity between the modeled signal before artifact inclusion and after FilterBlink application, with a logarithm fashion (p < 0.001 comparing waves for L = 0.1 and L > 0.6, Wilcoxon Rank Test, for Fz, Pz, and Oz, Fig 4B).

**Fig 5:**
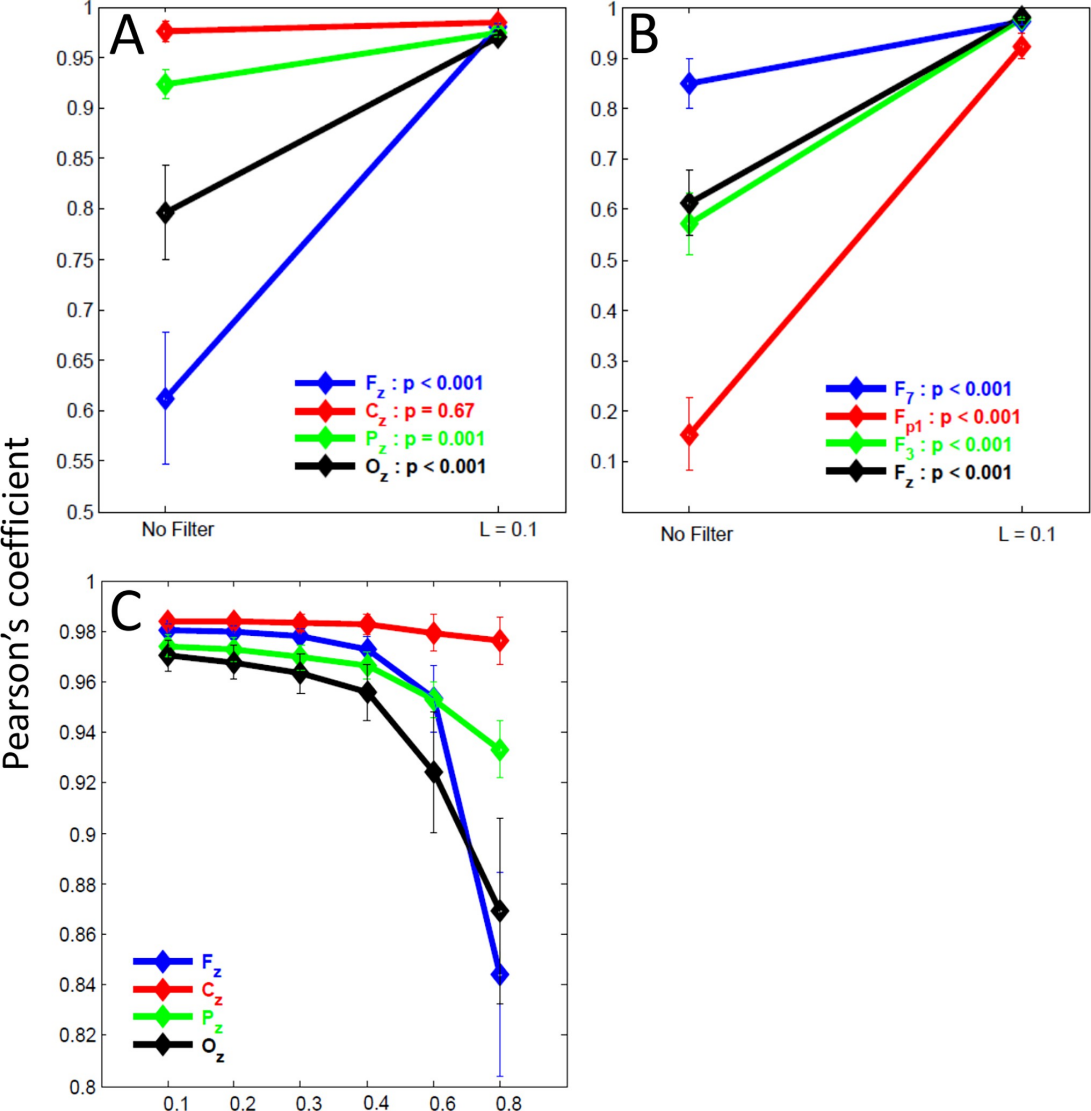
Analysis of the FilterBlink effect on the correlation index between modeled ERPs without VEOGs and modeled ERPs with VEOGs in the ongoing EEG. (a) Correlation index prior to and after FilterBlink application (L = 0.1). (b) Correlation index for different L values (sample mean ± std. dev.).

As observed for the ongoing modeled EEG signal, the sinusoidal ERPs were unaffected either by artifact inclusion or by the application of FilterBlink at Cz (Fig 5A, red line). However, FilterBlink recovered the sinusoidal wave overlaid on the blink and preserved the remaining artificial ERPs (before 500ms in the epochs).

**Fig 6:**
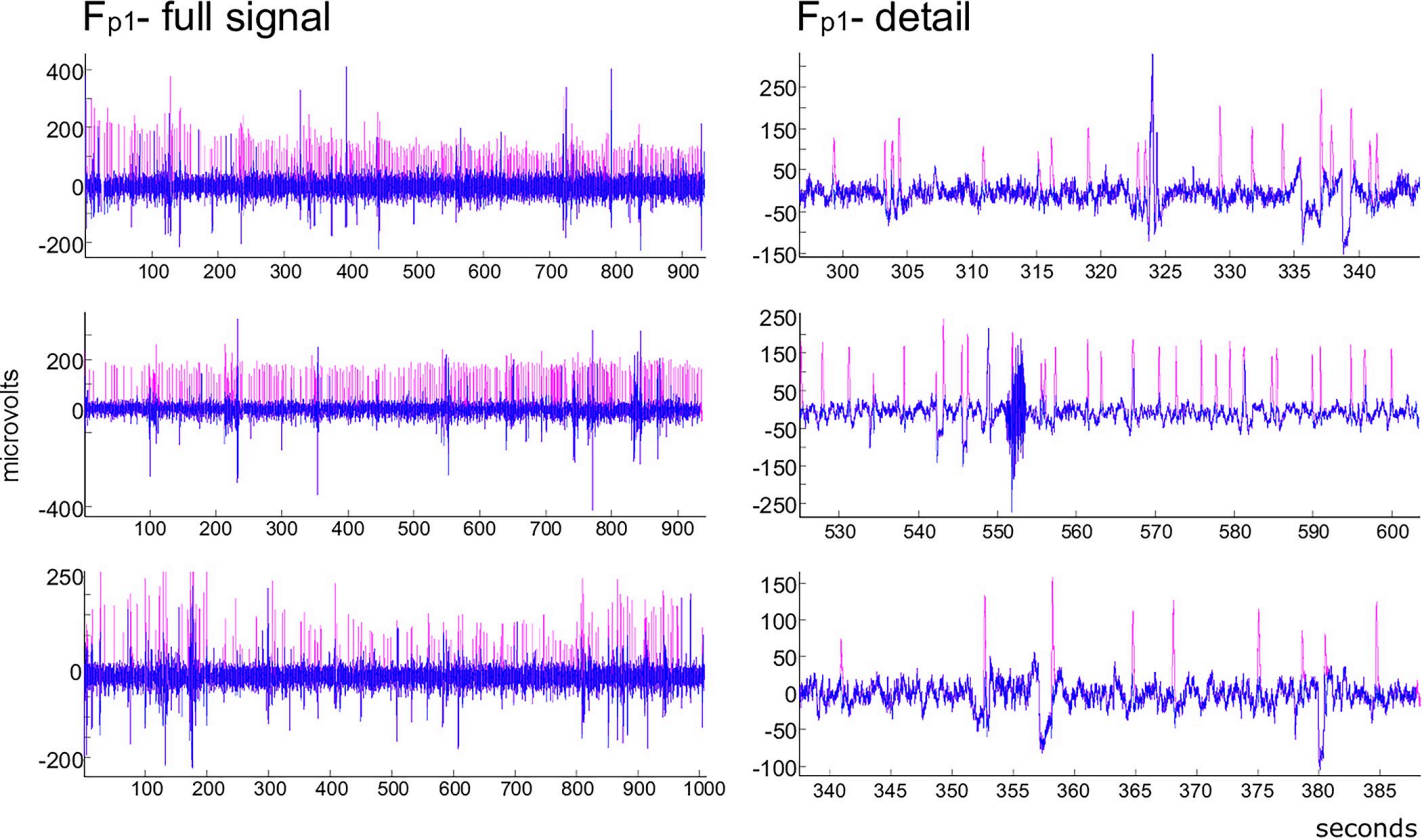
Real ongoing EEG signal prior to (magenta) and after (blue) FilterBlink application. Samples from the Fp1 channel, for three subjects, filter applied using L = 0.1.

Comparing Pearson’s coefficients of the correlation between artificial ERPs before and after FilterBlink, we observed that signal recovery was confirmed, which showed a significant change from r = 0.15 to 0.9 in the Fp1 channel, or a more discrete effect, changing from r = 0.92 to 0.97 in the Pz channel, (see Fig 5A and 5B, p < 0.001 comparing Pearson’s coefficients prior to and after FilterBlink application for L = 0.1). The effect of FilterBlink is more substantial on anterior channels (see histograms of the coefficient differences before and after applying FilterBlink for all modeled channels in Fig 6, showing the magnitude of the FilterBlink effect on whole scalp signals). Similar to the ongoing modeled signal, the increase in L value reduces signal recovery in a logarithmic curve. Pearson’s coefficients differed statistically from L = 0.1 and L > 0.6 for Fz, Pz, and Oz (p < 0.01, Fig 5B).

Artifact suppression occurred in the biological ongoing EEG from behavioral testing in the Fp1 signals for three different subjects prior to (magenta) and after (blue) FilterBlink application (Fig 6, for L = 0.1). In a closer view (Fig 6, right column), the EEG background was unaffected by the method while the blink artifacts were subtracted, and some non-linear residual oscillation remains.

**Fig 7:**
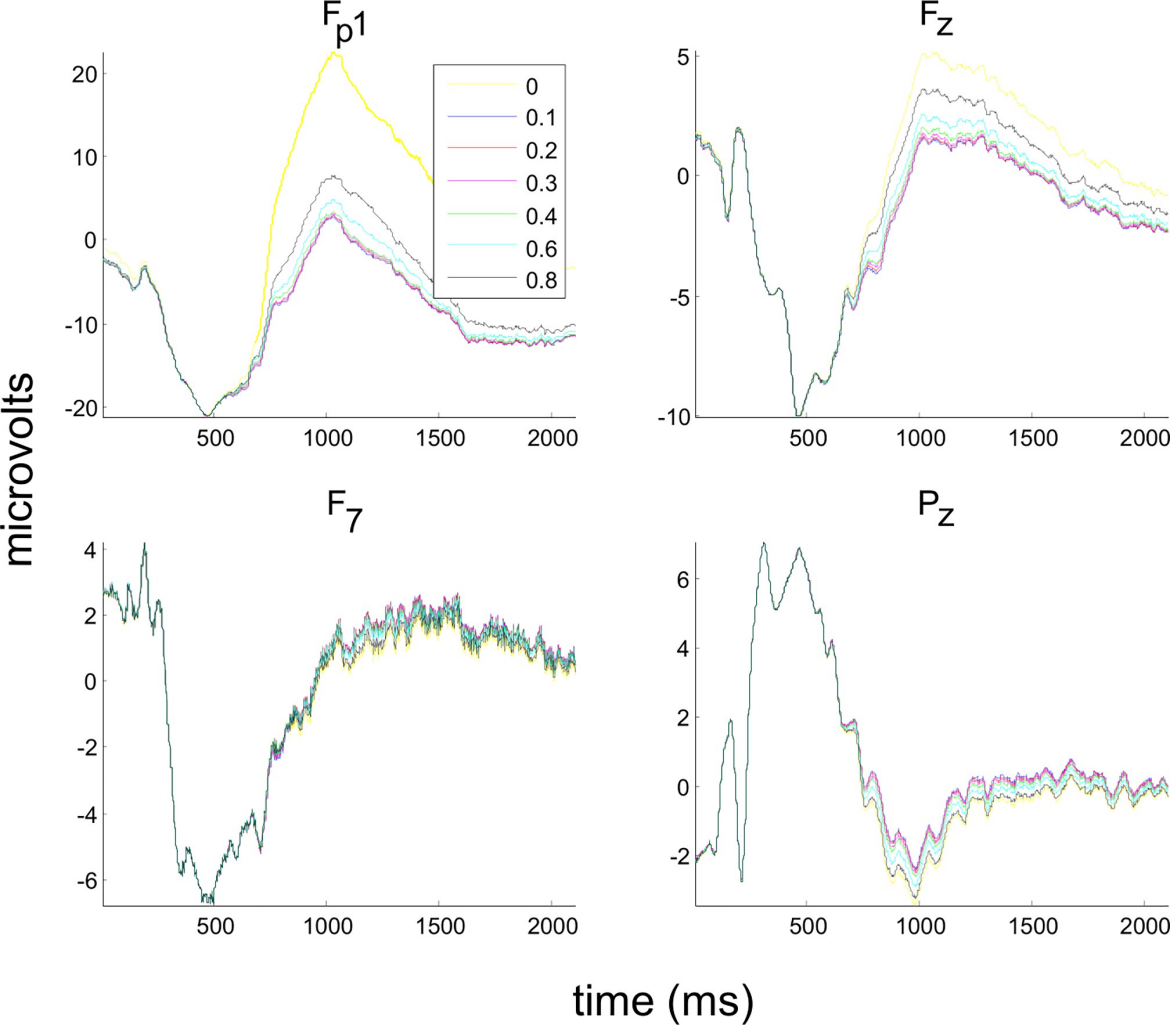
Application of FilterBlink on a real EEG signal and subsequent ERP study. Inbox, *L* values for filtering, that are indicated by respective colored line.

After grand averaging, the ERP wave characteristics can be identified in the channels of interest (in Fig 7, Fp1, F7, Fz, and Pz). Also, at the averaged VEOGs (seen in Fig 7, yellow wave), the ERPs are restricted to the first 1000 ms in the epochs while the amplitude of the blink artifact increases after that time. The effect of FilterBlink application is more pronounced up to L = 0.4.

**Fig 8:**
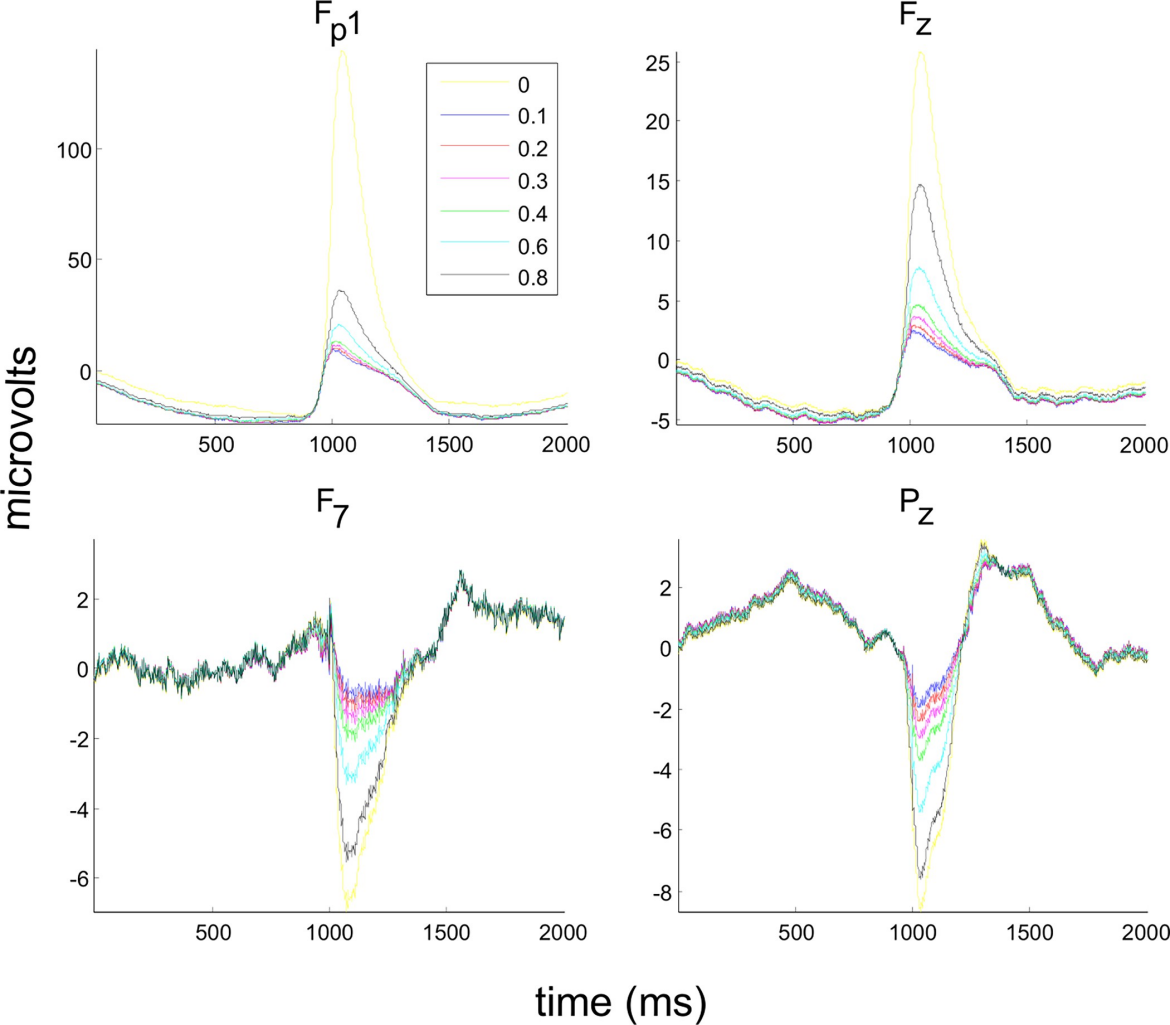
Application of FilterBlink on a real EEG signal and subsequent promediation centered on VEOG detected positions. Inbox and axis as in Fig 7.

The averaged VEOG positions (Fig 8) confirm the suppression of blink artifacts up to L < 0.6, where the voltage is near the zero line. However, a slight positivity/negativity remains after the suppressions in all illustrated channels.

Removing artifacts is an important task for ERP analysis when such elements are relatively synchronized to the signals of interest. The main purpose of FilterBlink has been to improve the quality of ERPs, which can be degraded by blink artifacts relatively locked to them. The averaging approach is here applied in reverse: VEOG templates obtained with EEG averaging at the positions where the blink artifacts were detected (from frontopolar channels, with the greatest VEOG amplitude) are subtracted from these very time positions; this suppresses only the patterned portion of the artifact, and the solely remaining part is non-linear variations, which naturally disappear with ERP averaging.

## 4. Discussion

The present method is notably more straightforward than ICA and more conservative than the correction method by regression techniques since FilterBlink acts locally on the EEG signal and does not subtract neural information [4]. We summarize in Table 1 some relevant points of methodologies to artifact suppression compared with the most used method nowadays, Independent Component Analysis (ICA).

**Table 1:** Qualitative comparison between FilterBlink and ICA.

Parameter	FilterBlink	ICA
loss of information	Minimal and local	Wide and larger as lesser number of components (i.e., number of channels)
Algorithm and Theory	Very simple	Very complex
Source modelling (VEOG)	Detection and averaging	Probabilistic estimation
Use	Only Event-Related Potentials (by averaging)	Time (transient potentials) and frequency domains.
Minimal requirement	Two channels to VEOG detection	More sources than the number of channels
Number of EEG channels	Any	As many as possible (minimizing loss of information)
restrictive characteristics of the EEG	None	Non-gaussian distribution

As used to study brain function, ICA assumes that the EEG signal captured in each derivation results from several distinct, independent brain sources, which are mixed and recorded at each electrode site. Thus, mathematically, it would be possible to infer what these independent sources would be by comparatively evaluating the behavior of the EEG in the different derivations. However, the estimated sources are adjusted into a number of components equal to the analyzed derivations. In other words, an estimated component does not correspond to a single source but rather to a summation of other ones with different weights. Therefore, the more channels, the greater the chance of isolating specific sources within the components. To understand brain physiology, ICA is a fruitful path. However, with 20 channels, it is reasonable to assume that a component does not just contain the signal from only one source. Therefore, ICA is also the most common method for extracting artifacts by subtracting the components where VEOG or muscle activity is expressed from the integral signal. Logically, with only 20 channels, we would be suppressing some relevant brain activity when we eliminate the VEOG/muscle component from the EEG signal because the number of electrical sources is not less than the number of channels [24, 26]. The proof for this assumption is to observe that ICA somehow distorts the signal at time instants in which there is no blink, where there is no reasonable evidence to assume such kind of artifact presence. Observing data before and after VEOG removal, it is evidenced that ICA seems to distort the EEG signal outside the blinking artifact window time in low-density EEG recording [27–29]. Furthermore, ICA may not even work in setups with fewer electrodes [30]. Thus, our major concern is that the ICA could corrupt the signal for EEG setups with fewer channels by carrying neural information in the artifact components since there are many more sources than receptors [24]. FilterBlink is a very conservative method because it minimally corrupts the ongoing EEG since the intervention is punctual over a minimal time segment, centered on the positions where the VEOGs were detected, with the same size as the lower portion of the template epoch (only the VEOG deflection, detected between the two minima around the center of the template, see Fig 1) used in the process. The worst outcome of the Filterblink application is a lesser suppressive effect over VEOGs than desired.

However, our method proved significantly effective: it is controlled and applied in the time domain using statistically obtained real elements without any further signal handling (such as decomposition), and its results could be regarded as predictable and reliable. FilterBlink over the EEG model recovered nearly 98% of the original ongoing signal, and the same result was observed for artificial ERPs, which were partially overlaid by the VEOGs. The model should be regarded as consistent and representative since the biological EEG signal from the mid-central channel of each subject (with the lowest VEOG artifact and with the main brain rhythms [31]) was used, as well as the respective subjects’ VEOG templates for each channel, which are also biological, real elements. The artificial sinusoidal wave for ERP emulation, with low amplitude, was a known pattern for accurately evaluating the method after promediation.

As a matter of fact, there are statistical approaches for algorithm processing, obviously because the EEG signal is a complex dataset with chaotic behavior. However, estimates are restricted to detecting VEOGs, which are performed by marking points on the wave with amplitude values greater than *k* standard deviations. We have set EEG epochs by a time window centered in such detected positions. With epoch averaging, blink templates are obtained. These templates reveal the stable VEOG pattern since, as in the study of ERPs, nonlinearities in the EEG signal are eliminated through averaging. Thus, by axiom, these templates are the best possible approximation of the real electrophysiological activity derived from the vertical movement of the retinal electrical vector, recorded in superimposition to the EEG, mainly in the frontal derivations. By subtracting this template from the EEG segments from which it was extracted, we consequently obtain the EEG segment that comes closest to the ideal suppression of the VEOG artifact. VEOG are different from other electrographic phenomena, as the nonlinearity of the EEG does not hide them, and their source is known. Therefore, we do not need to estimate it using methods such as ICA. Just detect it using a simple statistic (standard deviations from the signal average), extract its pattern (template), and suppress this pattern from the original signal without affecting the EEG as a whole. Suppose the signal is previously treated to eliminate other artifacts with large amplitude and filtered for long-range oscillations with a frequency smaller than 0.5Hz. In that case, the error tends to be minimal if an adequate value for *k* is chosen (1.5 standard deviation seems suitable).

The *L* parameter (the threshold of similarity applied to Pearson’s test coefficient regarding the correlation between the template and the actual EEG segment) is chosen according to the user’s objectives. Once these parameters are defined, we can select if FilterBlink will be more or less conservative, respectively, losing some VEOG events in the blink detection or detecting other EEG elements with smaller amplitude and correlation with the template. In other words, a threshold of similarity between the template and EEG segment (given by L) allows the user to set the sensitivity and specificity of the filter. In this case, the lowest value (*L* = 0.1) was the most effective for the model, although the signals remained stable only up to 0.6.

The non-linear VEOG residues that remain in the ongoing EEG after FilterBlink use limit the application of the present method for ERP analysis in the time domain as these residues would yield a persistent low-frequency artifact for the power analysis at the frequency domain in the anterior channels, similar to the original VEOG spectra. Thus, the suitable use for FilterBlink would be restricted to averaging EEG signals, such as in an ERP study. It is difficult to say how much this "residue" is not a positive or negative long latency ERP that becomes evident with VEOG averaging. Prior to the VEOG averaged positions, a negative/positive deflection is observed, probably related (at least in part) to the ERPs that frequently occur right before the blinking.

## 5. Conclusion

While ICA, the most used method for artifact suppression, can distort the brain signal when working with few EEG leads, the FilterBlink method constitutes a more conservative alternative since it keeps the signal between blinks unaltered. Among possible solutions, the simplest may be the best to paraphrase Occam’s Razor Principle. Thus, FilterBlink is an intuitive and simple solution whose efficiency has been predicted, and the results with real signals have confirmed it. Thus, in situations where blinks may represent an issue, FilterBlink may be useful in analyzing ERP signals instead of ones in the frequency domain, given the residual of VEOG artifacts.

## Supporting information

S1 File (DOCX)
