# Supplementary material for: Conservative method for vertical electrooculogram attenuation based on local suppression of ongoing EEG artifact templates
Source: PLoS One. 2024 Jul 18;19(7):e0305902. doi: 10.1371/journal.pone.0305902 (PMC11257361; doi:10.1371/journal.pone.0305902)
Supplement: S1 File — (DOCX) [file pone.0305902.s001.docx]

function y = filter_blink(x, n, L ,k, c)

% y = filter_blink(x, n, L ,k, c)

%

% INPUT: x = 2-D array (channels vs bins) where rows 4 and 12 are Fp1 amnd Fp2 channels

% (for rule, Fp1 and Fp2 leads),

% n = sensivity to blink triggering (number of standart deviations from

% baseline signal to detect events). Default is 1.5 SD

% r = correlation index. Default is 0.2

% k = epoch size (in bins). Default is 640sec * sampling_frequence

% c = coincidence threshold (rate of detected blinks between Fp1 and Fp2)

% Default is 0.85.

% plot = value diff of zero: plot processing stages (default is 0).

%

% OUTPUT y = filtered signal for all channels

%

% General instructions: the Filter_Blink is a supervised method and the EEG

% signal should be visually inspected before Filter_Blink application, for

% several artifact removal (such as high amplitude miograms) which will

% bbias the sensivity "n".

% The parameters can be manipulated for more or less sensitivity and

% specificity as operator desire.

% We Strongly recomend post visual inspection of EEG signal of Fp1 and Fp2

% channels superimposing pre- and post-filtering to qualitatively observe

% method efficacy.

if nargin < 6,

if_plot=0; end

if nargin < 5,

c = 0.85; end

if nargin < 4,

k = 400; end

if nargin < 3,

L = 0.6; end

if nargin < 2,

n = 1.5; end

chs = {'Fp1', 'Fp2', 'Fz', 'Cz', 'Pz', 'O1', 'O2'}; % for conventional 10-20 system

trig1 = x(4,:);

trig2 = x(12,:);

trig1_std = std(x(4,:)).*n;

trig2_std = std(x(12,:)).*n;

tt1 = find(trig1>(trig1_std+mean(abs(trig1))));

tt2 = find(trig2>(trig2_std+mean(abs(trig2))));

tt_pos1 = find(diff(tt1)>k/2)+1; %separa os grupos de pontos de cada

tt_pos2 = find(diff(tt2)>k/2)+1; %piscada

t1 = tt1(tt_pos1);

t2 = tt2(tt_pos2);

v1 = zeros(1, length(trig1)); v1(:) = NaN; v2 = v1;

v1(t1) = 1;

v2(t2) = 1;

Vs = sum(v1==v2);

Cs = (min([length(t1) length(t2)])/max([length(t1) length(t2)]));

disp([' F_p_2 <> F_p_1; coincidencia quantitativa = ' num2str(Cs*100) '%'])

disp([' F_p_1; coincidencia topografica = ' num2str(Vs/length(t1)) '%'])

disp([' F_p_2; coincidencia topografica = ' num2str(Vs/length(t2)) '%'])

%%%%%%%%%%%% -------------------------------------

if Cs > c,

if length(t1) < length(t2), tt=t1; else, tt=t2; end

disp([' Numero de blinks = ' num2str(length(tt))])

k = round(k/2);

if (tt(end)+k) > length(x), tt=tt(1:end-1); end

all_pts = zeros(length(tt), 2*k+1);

for i=1:length(tt),

all_pts(i,:)=tt(i)-k:tt(i)+ k; % 'trigger + interval' array

end

ccv=zeros(20,(k*2)+1);

for ch = 1: 20, %cycle through channels

xc=x(ch,:);

ccv(ch,:) = mean(xc(all_pts)); % mean channel vector (ERP)

end

clear xc

v = mean(abs(ccv));

ep(1) = find(v == min(v(1:length(v)/2)));

ep(2) = find(v == min(v(length(v)/2:end)));

if if_plot~=0,

figure

plot(ccv'),

hold on

plot([ep(1) ep(1)], [min(ccv(:)/2) max(ccv(:)/2)],'k--')

plot([ep(2) ep(2)], [min(ccv(:)/2) max(ccv(:)/2)],'k--')

end

% extracting event from background signal...

bn=zeros(1,20);

y=x;

for ch = 1:20,

m = ccv(ch,ep(1):ep(2));

for i = tt,

s = x(ch, (i-k)+ep(1):(i-k)+ep(2));

r=corrcoef(s,m);

if r(2) >=L,

y(ch, (i-k)+ep(1):(i-k)+ep(2))=s-m;

bn(ch)=bn(ch)+1;

end

end

end

disp([' F.B. > Percent of exclusion "Ex" of detected events per channel (INT%)'])

for ch=[4 12 17 18 19 8 16]

disp([' + channel # ' num2str(ch) '; Ex =' num2str(fix(bn(ch)*100/length(tt))) ' %']);

end

else

disp('Number of coincidences between Fp1 and Fp2 is bellow the threshold. Aborted operation')

y = 0;

endfunction
